# Perceptions of Artificial Intelligence Among Healthcare Staff: A Qualitative Survey Study

**DOI:** 10.3389/frai.2020.578983

**Published:** 2020-10-21

**Authors:** Simone Castagno, Mohamed Khalifa

**Affiliations:** Department of Interventional Radiology, Royal Free Hospital, London, United Kingdom

**Keywords:** artificial intelligence, healthcare, survey, questionnaire, online, perceptions, attitudes, public health

## Abstract

**Objectives:** The medical community is in agreement that artificial intelligence (AI) will have a radical impact on patient care in the near future. The purpose of this study is to assess the awareness of AI technologies among health professionals and to investigate their perceptions toward AI applications in medicine.

**Design:** A web-based Google Forms survey was distributed via the Royal Free London NHS Foundation Trust e-newsletter.

**Setting:** Only staff working at the NHS Foundation Trust received an invitation to complete the online questionnaire.

**Participants:** 98 healthcare professionals out of 7,538 (response rate 1.3%; CI 95%; margin of error 9.64%) completed the survey, including medical doctors, nurses, therapists, managers, and others.

**Primary outcome:** To investigate the prior knowledge of health professionals on the subject of AI as well as their attitudes and worries about its current and future applications.

**Results:** 64% of respondents reported never coming across applications of AI in their work and 87% did not know the difference between machine learning and deep learning, although 50% knew at least one of the two terms. Furthermore, only 5% stated using speech recognition or transcription applications on a daily basis, while 63% never utilize them. 80% of participants believed there may be serious privacy issues associated with the use of AI and 40% considered AI to be potentially even more dangerous than nuclear weapons. However, 79% also believed AI could be useful or extremely useful in their field of work and only 10% were worried AI will replace them at their job.

**Conclusions:** Despite agreeing on the usefulness of AI in the medical field, most health professionals lack a full understanding of the principles of AI and are worried about potential consequences of its widespread use in clinical practice. The cooperation of healthcare workers is crucial for the integration of AI into clinical practice and without it the NHS may miss out on an exceptionally rewarding opportunity. This highlights the need for better education and clear regulatory frameworks.

## Introduction

Artificial intelligence (AI), described as the ability of a digital computer to perform tasks commonly associated with intelligent beings (Copeland, 2020), is not a new concept. Alan Turing first asked the question “Can machines think?” in his famous paper Computing Machinery and Intelligence (Turing, 1950) in 1950. However, in recent years the field of AI has seen a dramatic development thanks to advances in machine learning techniques as well as the availability of massive datasets, or “big data,” which has led to AI applications being increasingly prevalent in society and becoming an intrinsic part of our everyday lives (Laï et al., 2020). Some examples are Amazon’s product recommendation system for online shopping, ridesharing apps like Uber or Lyft and smart personal assistants such as Cortana, Alexa and Siri.

AI technologies are already being applied in healthcare, with the potential to profoundly transform medical practice and patient care. Possibly the most successful domain of medical AI applications is that of AI-assisted analysis of radiological images (Yu et al., 2018), which utilizes deep learning (a specialized subset of machine learning that uses neural networks to learn from unstructured data) to recognize disease patterns that could be missed even by experts. For example, a paper published on Nature shows that an AI system could outperform radiologists in the detection of breast cancer in mammograms (McKinney et al., 2020), while very recently an international team developed a diagnostic capable of predicting whether a patient is likely to have COVID-19 based on their symptoms (Menni et al., 2020).

Despite these positive initial results, there is still a lot of controversy and confusion on the subject of AI and its applications, with the public and even the scientific community being divided on its potential benefits and risks. While on one end of the spectrum the most skeptical are dubious about the actual capabilities of AI, on the opposite end some (including the late Stephen Hawking) are worried AI may eventually surpass human intelligence and become uncontrollable (Hawking et al., 2014). In the medical field, there are concerns that machine learning may lead to physician deskilling (Cabitza et al., 2017) and cause a distortion of the doctor-patient relationship (Karches, 2018). However, such concerns are often not specific to AI or machine learning, but rather on the way they are employed and therefore other authors believe that an appropriate, informed use of AI may be beneficial and may greatly improve patient care (McDonald et al., 2017; EsteChanva et al., 2019; Liyanage et al., 2019).

The purpose of this study is to assess the awareness of AI programmes among staff working at the Royal Free London NHS Foundation Trust and to investigate their perceptions toward AI applications in healthcare.

To the best of our knowledge, this is the first survey on the attitudes of health professionals toward AI in the NHS and one of the first in the world (Codari et al., 2019; Oh et al., 2019; Laï et al., 2020).

## Materials and Methods

We investigated the prior knowledge and opinions on the subject of AI of a variety of health professionals at the Royal Free London NHS Foundation Trust using an online survey.

### Participants

An electronic questionnaire on the perceptions of AI in healthcare was developed using the open-source “Google Forms” platform and was distributed to all 7,538 members of staff (Royal Free London NHS Foundation Trust, 2019) at the Royal Free London NHS Foundation Trust via the trust e-newsletter using a unique link to the online survey. Participation was voluntary and participants were informed about the goal of the survey in the preface of the questionnaire. All responses were anonymous and participants could not be identified from the material presented. Responses were not recorded unless the “submit” button at the end of the questionnaire was pressed and only one submission per participant was allowed. Informed consent was implied once the “submit” button was pressed. As the study does not involve vulnerable subjects and the risks of informational or psychological harm are minimal, ethical oversight from an Ethical Review Board was deemed not to be necessary (Whicher and Wu, 2015).

### Survey

The survey (Table 1) is in accordance with the Checklist for Reporting Results of Internet E-Surveys (CHERRIES) (Eysenbach, 2004) and includes a partially categorized question investigating the profession of each respondent (Q1) and seven closed-ended questions aimed at qualitatively assessing the prior knowledge of healthcare staff on the subject of AI (Q2–4) as well as their attitudes and worries about its current and future applications (Q5–8). Question Q5 references a public talk by Elon Musk at the 2018 SXSW Film Festival in Austin, TX, in which he described AI as far more dangerous than nuclear weapons (Musk, 2018). In question Q7, we use the word “useful” without further clarification for two main reasons: firstly, because we were interested in understanding the perceptions of healthcare professional toward not only current but also future AI applications. Although people with no prior knowledge of AI may have unrealistic views of how this technology will be employed in the medical field, they are likely to still carry positive or negative expectations toward its future use. Secondly, the word “useful” has been used in the same context in previous studies, such as in Oh’s article on the attitudes of Korean physicians toward AI (Oh et al., 2019).

**TABLE 1:** Online questionnaire on the perceptions of AI within health professionals. The survey was divided into three sections: Profession, knowledgebase, and Attitudes.

	Question	Answers
Profession	Q1. What is your profession?	Medical doctor
		Nurse
		Therapist
		Physician associate
		Manager
		Other: open text
Knowledgebase	Q2. How many applications of AI have you come across in your work?	None
		One
		Two to four
		More than four
	Q3. Do you know the difference between machine learning and deep learning?	Not at all
		I only know one term
		I know both terms but the difference is not clear to me
		I know both terms and the difference is clear to me
	Q4. How often do you use speech recognition or transcription applications?	Never
		Rarely
		Weekly
		On a daily basis
Attitudes	Q5. Do you think there may be serious privacy issues with the use of AI?	Completely agree
		Partially agree
		Partially disagree
		Completely disagree
	Q6. How much do you agree with the following statement: “AI is more dangerous than nuclear weapons” (Musk, 2018)	Completely agree
		Partially agree
		Partially disagree
		Completely disagree
	Q7. How useful do you think AI could be in your area of work?	Extremely useful
		Useful
		Of limited use
		Of no use at all
	Q8. How worried are you that AI will replace you at your job?	Extremely worried
		Moderately worried
		Mildly worried
		Not worried at all

AI, artificial intelligence.

### Statistical Analysis

The results obtained were analyzed using basic statistics (such as total numbers and percentages) and a subgroup analysis was performed using Kruskal-Wallis test followed by *post-hoc* pairwise Mann-Whitney U tests with Bonferroni correction for multiple tests in order to investigate variances in knowledge and attitudes within different healthcare professions (doctor, nurse, therapist, manager or other). The tests were performed using the Social Science Statistics calculators (Social Science Statistics). For all tests, the level of significance was set at *p*-value ≤ 0.05.

## Results

A total of 98 healthcare workers out of 7,538 (response rate 1.3%; CI 95%; margin of error 9.64%) completed the survey, of whom 34 were medical doctors, 23 nurses, 11 managers, seven therapists, and 23 other professionals (Table 2).

**TABLE 2:** Various professions of survey participants.

Profession	Number of respondents
Medical doctor	34
Nurse	23
Therapist	7
Manager	11
Other	23
Physician associate	8
Clinical researcher	6
Pharmacist	2
Patient flow coordinator	2
Lawyer	1
Technician	1
Assistant	1
Childcare	1
Support service	1
Total	98

**FIGURE 1:**
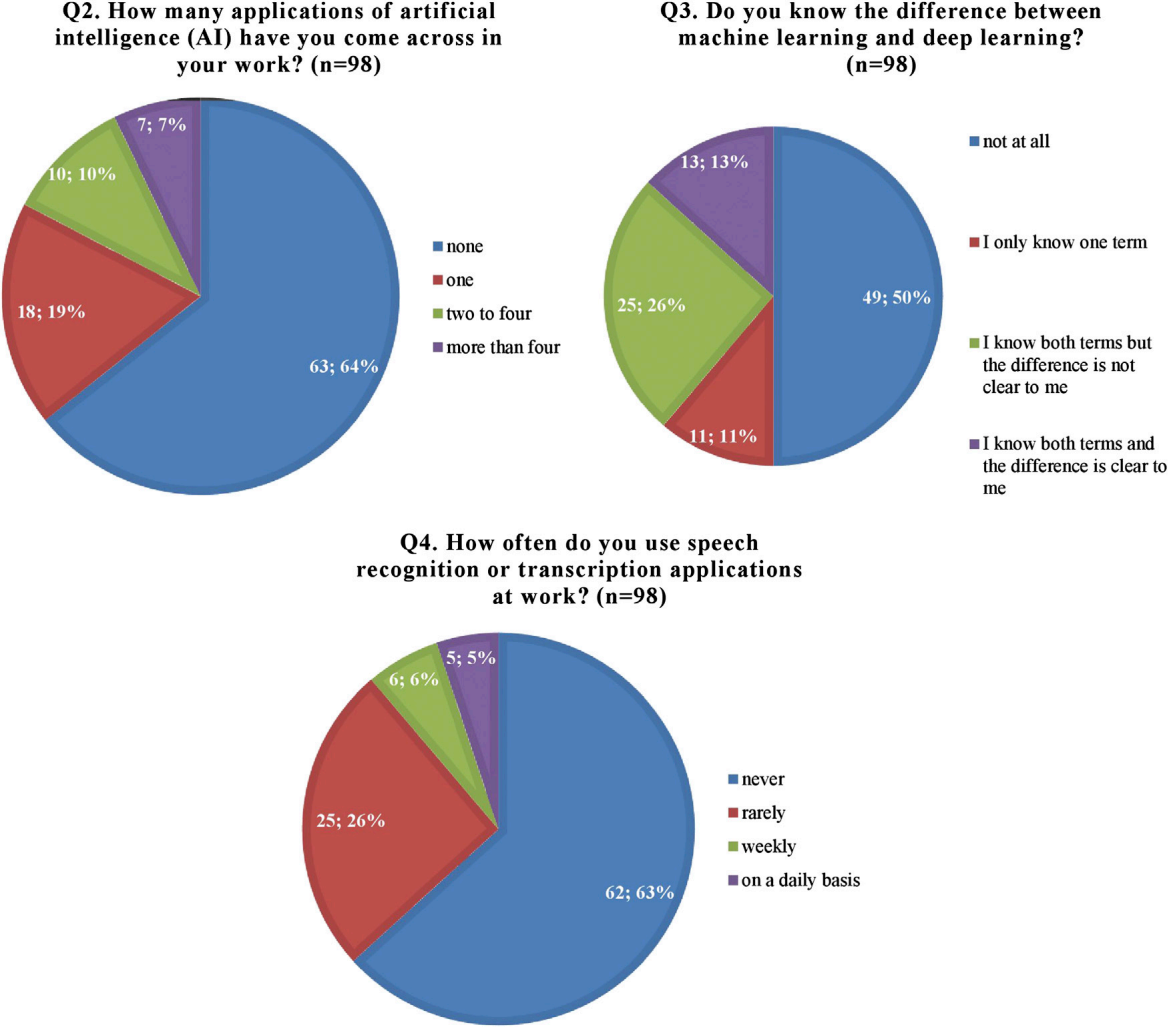
Participants’ knowledgebase regarding artificial intelligence (AI) and its applications.

In the “knowledgebase” section of the survey, almost two thirds of respondents (63, 64%) reported they had never come across applications of AI in their work and a remarkable 87% did not know the difference between machine learning and deep learning, although 50% knew at least one of the two terms. Furthermore, only 5% stated using speech recognition or transcription applications at work on a daily basis, while 63% never utilize them (Figure 1).

**FIGURE 2:**
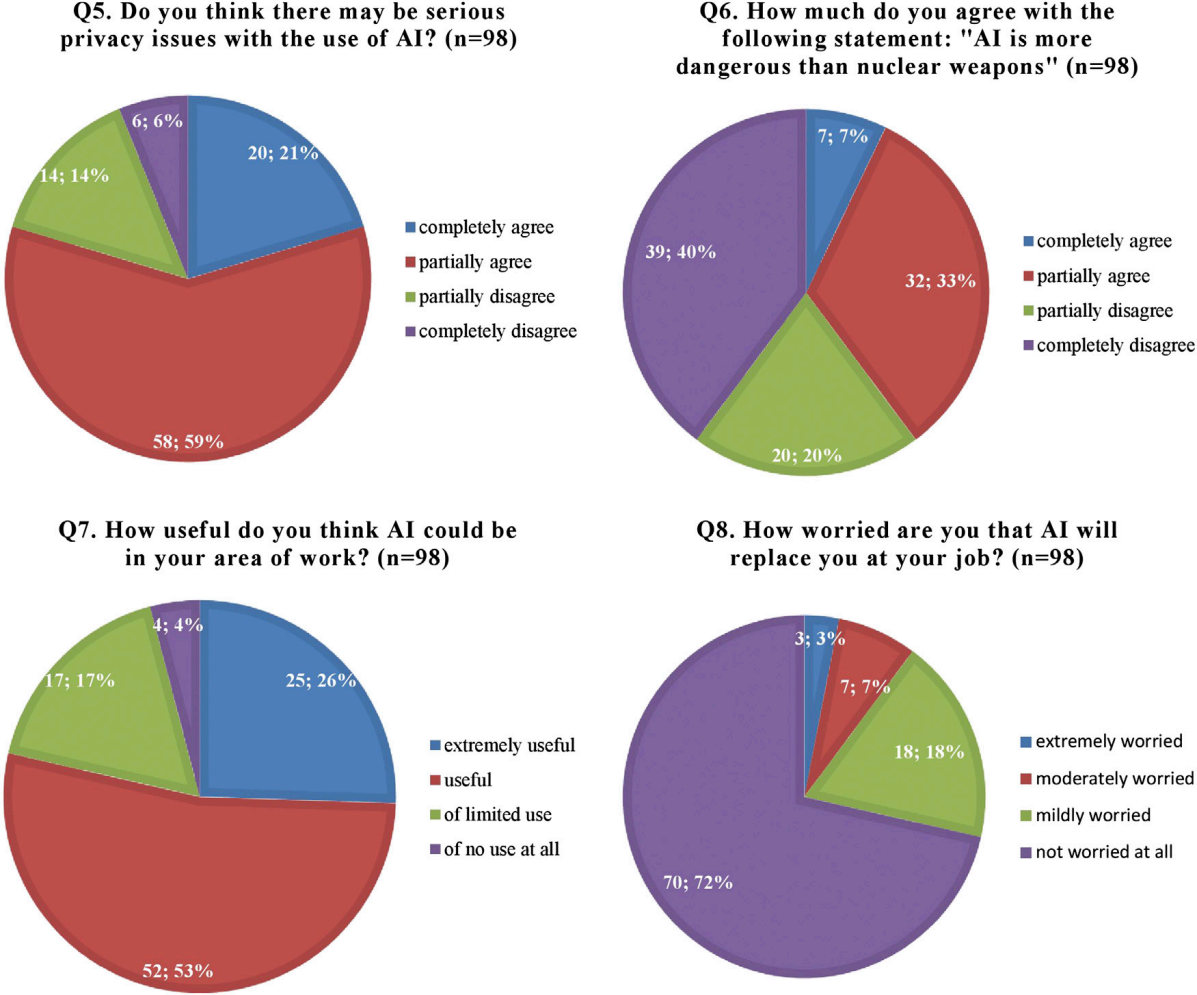
Participants’ attitudes and worries regarding artificial intelligence (AI) and its applications.

When investigating the attitudes of healthcare staff toward AI, the vast majority of respondents (78, 80%) believed there may be serious privacy issues associated with the use of AI and 40% considered AI to be potentially even more dangerous than nuclear weapons. However, most participants (77, 79%) also believed AI could be useful or extremely useful in their field of work and only 10% were worried AI will replace them at their job (Figure 2).

**FIGURE 3:**
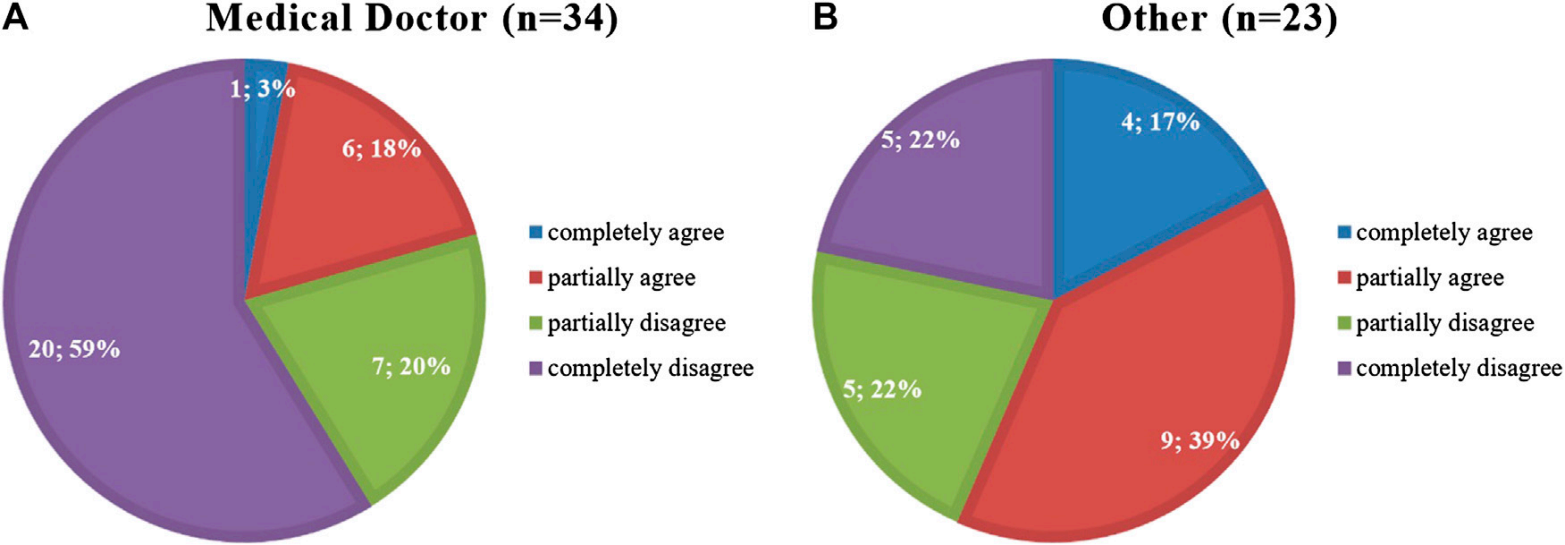
Results of Q6. How much do you agree with the following statement: “AI is more dangerous than nuclear weapons” (Musk, 2018) for the professions of **(A)** “Medical Doctor” and **(B)** “Other.”

These results were evaluated using a subgroup analysis (Table 3), which demonstrated significant differences according to profession only for three questions (Q5–7). Therefore, a *post-hoc* analysis for Q5–7 was conducted using pairwise Mann-Whitney U tests with Bonferroni adjustment to correct for multiple tests (Table 4). For questions Q5 and Q7, our data is not sufficient to make statements on pairwise differences between professions; however, *post-hoc* analysis on question Q6 reveals a statistically significant difference between the professions of “medical doctor” and “other.” As a matter of fact, while doctors appear less worried about the potential threat of AI, with only 21% agreeing or completely agreeing with the statement “AI is more dangerous than nuclear weapons,” a much greater percentage of other health professionals (56%) deem it to be more dangerous (Figure 3).

**TABLE 3:** Subgroup analysis according to participants’ profession.

Question	*p*-value^a^
Q2. How many applications of AI have you come across in your work?	0.91
Q3. Do you know the difference between machine learning and deep learning?	0.58
Q4. How often do you use speech recognition or transcription applications?	0.57
Q5. Do you think there may be serious privacy issues with the use of AI?	0.02
Q6. How much do you agree with the following statement: “AI is more dangerous than nuclear weapons” (Musk, 2018)	0.007
Q7. How useful do you think AI could be in your area of work?	0.01
Q8. How worried are you that AI will replace you at your job?	0.82

AI, artificial intelligence.

a *p*-values were calculated using Kruskal-Wallis test.

**TABLE 4:** *Post-hoc* analysis for questions Q5-7 according to participants’ profession.

Question	*p*-value^a^	*Post-hoc* ^b^
Q5. Do you think there may be serious privacy issues with the use of AI?	0.02	No significant difference
Q6. How much do you agree with the following statement: “AI is more dangerous than nuclear weapons” (Musk 2018)	0.007	Significant difference between “medical doctor” and “other” (corrected *p*-value 0.03)
Q7. How useful do you think AI could be in your area of work?	0.01	No significant difference

a *p*-values were calculated using Kruskal-Wallis test.

b *Post-hoc* analysis was conducted using pairwise Mann-Whitney U tests with Bonferroni correction.

## Discussion

The results of this survey demonstrate a general lack of knowledge on the subject of AI and of awareness of its applications. Half of respondents did not know what machine learning and deep learning are and only 13% knew the difference between these two terms. This general lack of education on AI as well as a degree of confusion regarding what constitutes AI could also explain why, despite AI programs already being used extensively in everyday clinical practice (from electronic health records and electronic prescribing to automated ECG interpretation, for example), almost two thirds of participants reported never coming across AI in their work. Furthermore, although speech recognition and transcription applications such as Alexa or Siri are widely used in everyday life, 63% of participants reported never using them at work. This may reflect a certain resistance to change that is quite typical of healthcare (LeTourneau, 2004; Mareš, 2018) and may be secondary to a lack of clarity regarding who is to be held responsible in the event of an error caused by an AI tool (Codari et al., 2019; Laï et al., 2020), especially when there is not a full understanding of how that AI tool behaves.

From this survey it also transpires that the majority of participants considers AI to be useful in the medical field, which is consistent with previous studies (Codari et al., 2019; Oh et al., 2019; Laï et al., 2020). Nonetheless, there are undoubtedly concerns on the safety of AI, with 80% of respondents believing there may be privacy issues associated with the widespread use of AI in healthcare and 40% agreeing with Elon Musk’s statement that “AI is more dangerous than nuclear weapons” (Musk, 2018). It is to be noted, however, that only 21% of medical doctors agreed with this statement, as opposed to 56% of other healthcare workers (excluding nurses, therapists and managers).

Interestingly, 72% of participants denied any worry that AI will replace them at their job, which is in contrast with the findings of previous works on AI. For example, already in 2013 an Oxford study (Frey and Osborne, 2017) suggested 47% of United States jobs are at risk of “computerization” in the next few decades, while two surveys by the Pew Research Center in 2015 (Smith and Anderson, 2016) and 2017 (Smith and Anderson, 2017) determined that two thirds of Americans expect that within 50 years robots and computers will do much of the work currently done by humans and that 72% are worried about such a future. A possible explanation for such discrepancy is the belief that AI cannot replicate human emotions or express empathy and therefore cannot engage in the multi-layered interaction necessary to reassure patients and gain their trust (Krittanawong, 2018).

The literature on the perceptions of the general public toward medical AI is scarce. However, a recent article published on Nature (Tran et al., 2019) showed that, out of the 1,183 participants enrolled, only 50% believed that the development of AI in healthcare was an important opportunity and 11% even considered it a great danger for their care and privacy. In particular, patients were worried about the possible consequences of an unwanted replacement of humans by AI and only a minority were ready to integrate fully automated AI tools in their care. These results show a more pessimistic view of the general public toward medical AI compared to healthcare staff; however, they also highlight very similar concerns regarding safety and the quality of care delivered and provide an important cue for reflection on how to best integrate AI tools in clinical practice.

## Conclusions

In conclusion, although the healthcare community is starting to realize the potential of AI to radically improve patient care, AI applications are still not being integrated in medicine as fast as the technology has been advancing (Laï et al., 2020). This discordance is at least partly due to a resistance of medical workers to accept technologies that they do not understand, and in some cases even fear, and could end up being very costly for the NHS. As a matter of fact, the potential of AI to cut costs, improve treatment and increase accessibility to healthcare (Forbes Insights, 2019) is expected to be extremely rewarding. For instance, Accenture predicts that AI applications may potentially result in annual savings of $150 billion for the United States healthcare economy (Accenture, 2017). It is therefore evident there is a need to educate healthcare staff and the general public on the principles of AI as well as create regulatory frameworks to define the responsibilities of each stakeholder. Because of the complexity of the subject, however, further discussion and research are required: for example, once the COVID-19 emergency has passed, a questionnaire on a larger scale could better highlight discrepancies in attitudes between various health professionals and in a diverse range of working environments. It would be useful to include a larger number of researchers in the study, including researchers involved in AI projects as they are likely to have a greater knowledge of AI compared to other groups of healthcare professionals and their attitudes toward this technology may therefore differ significantly. Finally, as the topic of responsibility in AI is controversial, it would also be interesting to ask participants who they think should be responsible for the clinical outcomes of AI as well as what legal and ethical issues they believe this AI revolution will bring about.

## Limitations

Some limitations of our study should be noted. First, the relatively small sample size (98 participants) did not allow us to detect statistically significant differences in responses between professions, except for question Q6, where doctors appear to be less worried about the potential dangers of AI compared to other healthcare professionals. In order to encourage participation in the study, the survey was deliberately made short and simple to complete. However, there were no monetary incentives and the questionnaire was posted on the trust e-newsletter only once due to the breakout of the COVID-19 pandemic soon after, therefore limiting the response rate. Second, no data were recorded regarding the participants’ age and other demographic information, which may have revealed differences between groups. Furthermore, selection bias cannot be excluded, as respondents may have been more interested in AI and may have expressed more positive views compared to non-participants. Finally, the participants may not have been representative of healthcare workers in general, although the study did include various professions and backgrounds.

## Data Availability Statement

All datasets presented in this study are included in the article/ Supplementary Material.

## Author Contributions

SC: conceptualization, data curation, investigation, methodology, software, visualization, analysis, writing of original draft, review and editing. MK: conceptualization, methodology, analysis, supervision, review and editing.

## Conflict of Interest

The authors declare that the research was conducted in the absence of any commercial or financial relationships that could be construed as a potential conflict of interest.
