# Supplementary material for: Perceptions of Artificial Intelligence Among Healthcare Staff: A Qualitative Survey Study
Source: Front Artif Intell. 2020 Oct 21;3:578983. doi: 10.3389/frai.2020.578983 (PMC7861214; doi:10.3389/frai.2020.578983)
Supplement: Supplementary file 2 [file Table2_v1.DOCX]

Dear Prof. Hartung,

My name is Simone Castagno, I am an academic foundation doctor at the Royal Free Hospital (part of UCL Medical School) and a biomedical engineer from Imperial College London.

I am writing to submit our original research article entitled “Perceptions of artificial intelligence among healthcare staff: a qualitative survey study” for consideration by the “Frontiers in Artificial Intelligence” journal.

The purpose of this study is to investigate the awareness of and the attitudes towards AI applications among healthcare staff at the Royal Free Hospital in London by conducting an online survey. The questionnaire was completed by various health professionals and showed that, despite agreeing on the usefulness of AI in the medical field, most of them lack a full understanding of the principles of AI and are worried about the potential consequences of the widespread use of artificial intelligence. This may hinder the integration of AI tools in clinical practice and may result in the NHS missing out on an exceptionally rewarding opportunity.

To the best of our knowledge, this is the first study of this type in the NHS and one of the first in the world and we believe it fits the scope of the “Frontiers in Artificial Intelligence” journal.

We confirm that this manuscript has not been published elsewhere and is not under consideration by another journal.

All authors have approved the manuscript and agree with its submission to the “Frontiers in Artificial Intelligence” journal.

Please address all correspondence concerning this manuscript to me at s.castagno@nhs.net.

Thank you for your consideration of this manuscript.

Sincerely,

Simone Castagno
